# Restaurant-based intervention to facilitate healthy eating choices and the identification of allergenic foods at a family-oriented resort and a campground

**DOI:** 10.1186/s12889-017-4333-5

**Published:** 2017-05-05

**Authors:** Lucia Tarro, Magaly Aceves-Martins, Yolanda Tiñena, Joan Lluís Parisi, Xavier Blasi, Montse Giralt, Elisabet Llauradó, Rosa Solà

**Affiliations:** 1 Education and health promotion, Functional Nutrition, Oxidation and Cardiovascular Disease Research Group, Medicine and Surgery Department Facultat de Medicina i Ciències de la Salut, Universitat Rovira i Virgili, C/Sant Llorenç 21, 43201 Reus, Spain; 2 Eurecat-Reus, Centre Tecnològic de Catalunya, Avda Universitat 1, 43204 Reus, Spain; 3 Cambrils Park Resort and Camping Sangulí, C/ Passeig de Miramar s/n, 43840 Salou, Spain; 4 Unit of Pharmacobiology, Functional Nutrition, Oxidation and Cardiovascular Disease Research Group Facultat de Medicina i Ciències de la Salut, Universitat Rovira i Virgili, C/Sant Llorenç 21, 43201 Reus, Spain; 5 Unit of Lipids and Arteriosclerosis, Servei de Medicina Interna, Hospital Universitari Sant Joan de Reus, IISPV, Universitat Rovira i Virgili, CIBER Diabetes and Associated Metabolic Disorders (CIBERDEM), Reus, Spain

**Keywords:** Healthy menu choices, Health promotion, Health environment, Restaurant-based intervention

## Abstract

**Background:**

Restaurant-based interventions can be an enjoyable way to encourage healthier eating choices by all members of a family. Thus, the principal aims of this study were a) to promote healthy diets by increasing healthy food offerings and b) to increase the number of foods offered specifically as gluten-free and lactose-free and to inform patrons by including nutritional and allergen information that complies with *Regulation 1169/2011* regarding the food served in restaurants, takeaways and snack bars.

**Methods:**

A restaurant-based intervention was implemented at 16 food establishments at 2 resorts (the Cambrils Park Resort and Camping Sangulí, Spain, from 2014 to 2015) based on the following 4 components: 1) providing nutritional and allergen analyses of the offered dishes, 2) increasing the number of healthy food choices, 3) identifying menu items associated with allergies and intolerance, and 4) training staff on healthy eating and allergens. Customer satisfaction regarding food aspects was assessed using surveys (10-point scale).

**Results:**

Both resorts significantly increased their offerings of healthy dishes (28.6% to 44.7%; *P* = 0.003) and desserts with fruit (20% to 51.3%; *P* = 0.013), thus obtaining the Spanish Government’s Mediterranean Diet certification. Additionally, both resorts obtained Catalan Celiac Association certification. Moreover, both resorts significantly increased their percentages of gluten-free dishes (2.1% to 50.5%; *P* < 0.001) and lactose-free dishes (5.5% to 37.5%; *P* < 0.001) after the intervention. Customer satisfaction increased (mean ± standard deviation) from 6.9 ± 1.6 to 8.5 ± 1.5 (*P* < 0.001).

**Conclusion:**

This restaurant-based intervention expanded the number of healthy and allergen-free foods offered in a family-oriented holiday resort environment to encourage healthy food choices, resulting in increased customer satisfaction.

**Electronic supplementary material:**

The online version of this article (doi:10.1186/s12889-017-4333-5) contains supplementary material, which is available to authorized users.

## Background

In recent years, the frequency with which individuals dine outside their homes, particularly in restaurants, has increased [1–3]. A possible negative impact of this behaviour on nutritional habits has been described [4]. Eating in restaurants is associated with unhealthy food choices [5], weight gain [6] and obesity [7, 8]. In addition, compared with eating at home, dining out is considered a cause of the increased consumption of sweets and bakery products, soft drinks and other non-alcoholic beverages [2]. Thus, eating outside the home distances diners from the Mediterranean diet, which is considered beneficial and protective because of its high contents of fruits and vegetables, cereals, nuts and olive oil, which reduce the risk of cardiovascular disease and obesity [9].

The literature describes tools that restaurants can use to clarify menu labels to facilitate healthy eating (for example, by offering low-fat entrees and providing nutritional information) and increase the number of options for healthy eating (for example, by increasing the number of healthy or low-fat entrees as well as fruit and vegetable servings) [10]. In the U.S., a tool proposed by the Nutrition Environment Measures Survey in restaurants (NEMS-R) has the following aims: a) increase the number of healthy food choices (i.e., the accessibility of healthy options), b) expand the availability of specific foods (i.e., fruit, non-fried vegetables, and whole-grain bread) and beverages (diet soda and 100% fruit juice), and c) provide nutrition information to identify healthy food choices [11].

This tool proposes criteria similar to those of the Spanish Mediterranean Diet (AMED) certification for increasing the number of healthy food choices on restaurant menus to encourage healthier eating habits outside the home [12]. Thus, restaurant involvement in the creation of a healthy environment would involve offering dishes as an enjoyable way to improve customers’ health and fight obesity, cardiovascular diseases, and associated problems.

Recently, 6 restaurant-based strategies were proposed to improve the nutritional information provided to customers on menus to promote healthy choices [5]. In addition, based on a review of 27 restaurant interventions, national and international policy changes, such as obligatory nutritional menu-labelling in restaurants, were suggested [5].

However, the confirmation of the effects of these 6 restaurant-based strategies, such as the introduction of healthy choices and labels on restaurant menus [5], has been limited. To help restaurants promote healthy choices in an enjoyable family environment, robust evidence is required.

Additionally, the prevalence of food allergies has increased to 10.8% of adults [13] and 6–8% of children, affecting families around the world [14]. Food intolerance is common in the modern world, affecting 15–20% of adults [15]. The most common types of food intolerance worldwide involve certain cereal products (gluten), dairy products (lactose), certain vegetables, drinks (coffee) and miscellaneous items (hot spices) [15]. Consequently, avoiding restaurant dishes that could aggravate food intolerance is a challenge. Furthermore, in December 2014, the Spanish government approved the *1169/2011* menu-labelling regulation, which stipulates that restaurants must provide customers with information regarding the contents of 14 ingredients associated with allergies/intolerance, including gluten, crustaceans, eggs, fish, peanuts, soy, milk, nuts, celery, mustard, sesame, sulphites, lupin and shellfish, in all offered dishes [16]. These 14 foods are associated with food allergies and intolerance, which induce different responses in humans: allergies involve the immune system, while intolerance is a condition in which a food cannot be digested [15, 17]. Customers and their families need information on allergy- and intolerance-associated food to facilitate their choice of dishes. However, restaurant interventions that specify the process of implementing changes to respond to this demand are not available, leading to a challenge for restaurants.

Consequently, the Cambrils Park Resort and Camping Sangulí, two family-oriented holiday resorts with various types of food establishments, such as restaurants, takeaways and snack bars, are determined to encourage offering healthy foods and allergen-free dishes that are free of ingredients associated with sensitivity and allergies/intolerance (gluten and lactose). Thus, the principal aims of this study were a) to promote healthy diets by increasing healthy food offerings and b) to increase the number of foods offered specifically as gluten-free and lactose-free and to inform patrons by including nutritional and allergen information that complies with Regulation 1169/2011 regarding the food served in restaurants, takeaways and snack bars. A secondary aim was to improve customer food satisfaction.

## Methods

### Characteristics of the Cambrils Park resort and Camping Sangulí

The Cambrils Park Resort and Camping Sangulí together possess 16 food-service establishments. A common goal of the resorts was to improve food service quality for customers.

At the Cambrils Park Resort and Camping Sangulí, the food service was organized similarly. At both resorts, one quality manager developed the menus, and one chef oversaw the menus of the restaurants, the takeaways and the snack bars.

Since March 2014, the Cambrils Park Resort and Camping Sangulí had been supported by an external health promotion and education research team from Universitat Rovira i Virgili (URV) that is composed of 2 physicians and 3 nutritionists and whose aim is to direct intervention implementation.

### Intervention implementation at the Cambrils Park resort and Camping Sangulí

The intervention implementation period was from May 2014 to September 2015 at the Cambrils Park Resort (Cambrils, Spain) and Camping Sangulí (Salou, Spain). The intervention was based on 4 components: 1) providing nutritional and allergen analyses (*Regulation 1169/2011)* of the offered dishes; 2) increasing the number of healthy food choices, including dishes cooked with healthy foods, such as vegetables, whole grains and fruits and with healthy cooking methods, e.g., not fried or excessively salty; 3) identifying menu items associated with allergies and intolerance (14 sensitivity-associated ingredients regulated by a new national law) and providing choices for allergen- and sensitivity-free ingredients considering the most common triggers of allergies and intolerance (gluten and lactose); and 4) training staff on healthy eating and allergens (ingredients linked to allergies and intolerance). Moreover, customer satisfaction surveys on food aspects were developed.

This study was conducted in accordance with the Declaration of Helsinki and the International Conference on Harmonization Good Clinical Practice guidelines (ICH GCP). The Clinical Research Ethics Committee of the Institut d’Investigació Sanitaria Pere Virgili, the registered local ethic committee, determined that this project did not require their approval.

1. Analysis of nutritional and allergen contents
  1.1) The nutritional analysis of each dish followed the process described below:
    - First, both chefs compiled recipe cards that listed all of the ingredients used in each recipe and described the cooking instructions.
    - Second, the nutritional composition values (i.e., the macronutrient and micronutrient profiles) of each dish on the menu were determined using the PNC-Grams program® (Cesnid: Centre d’Ensenyament Superior de Nutrició i Dietètica, Barcelona, UB, Spain; 1.1 version, 2007) using the exact weight of each ingredient before preparation (raw) included in each recipe (listed on the card). Therefore, precise nutritional content information was available for all cooked dishes. Moreover, after the food was cooked, we calculated its oil absorption while taking into account the cooking technique (such as frying or grilling).
    - Third, the nutritional composition of the dishes was compared with the Dietary Reference Intake (DRI) of healthy middle-aged persons according to a Spanish reference [17]. For each dish, the percentages of the daily recommended values of energy (% of kcal), protein (% of g/d), total carbohydrates (% of g/d), sugar (% of g/d), total fat (% of g/d), saturated fat (% of g/d), and sodium (% of mg/d) were calculated.
  1.2) Allergen analysis of the offered dishes
    Using the recipe cards, we identified 14 allergens/sensitivity-associated ingredients according to *Regulation 1169/2011* regarding food allergies/intolerance and considering all of the ingredients in each dish offered in both resorts. The goal was to provide food choices for customers and their family members with allergies and/or intolerance.
2. Increase in the number of choices of healthy foods and allergen−/sensitivity-free ingredients associated with the most prevalent types of intolerance (gluten and lactose) in our environment
  We only focused on gluten and lactose because 1) they are the most commonly associated with intolerance [15] and 2) initiating cooking changes was easier for gluten- and lactose-free foods than for other allergy−/sensitivity-associated ingredients.
  2.1) Healthy food offerings according to AMED (Spanish government) recommendations
    In Spain, the Public Health Agency designed the AMED certificate [12] with the goal of maintaining and promoting the Mediterranean diet in the catering and restaurant environment and to accredit restaurants that accomplished specific goals.
  2.2) Allergen-free dish offerings
    2.2.1) Gluten-free dishes: Catalan Celiac Association (SMAP) certificate (Spain) recommendations
      In Catalonia (Spain), the Celiac Association designed the SMAP certificate for gluten-free food preparation with the aim of implementing appropriate systems for cooking without gluten cross-contamination in kitchens and restaurants to provide gluten-free dishes.
    2.2.2) Lactose-free dishes:
      In Catalonia (Spain), no regulations exist to assess whether restaurants offer lactose-free dishes. However, following the AMED criteria, changes were implemented to provide lactose-free dishes.
    2.2.3) Other allergens
      Dishes on the menus that contained allergens were identified with a specific logo but were not altered.
  2.3) Use of the 6 restaurant-based strategies described by Valdivia Espino et al. [5] to promote health through nutrition at the Cambrils Park Resort and Camping Sangulí:
    1. Increasing the availability of healthy options by adapting dishes in restaurants to be healthier;
    2. Enhancing the accessibility of healthy options by increasing the number of fruit and vegetable options, specifically in main dishes and desserts, while maintaining the price of each dish;
    3. Reducing prices to provide healthy and low-priced options;
    4. Informing consumers regarding the nutritional value of the dishes offered on restaurant menus;
    5. Designing restaurant policies regarding, for example, healthy food and healthy cooking techniques;
    6. Promoting and communicating healthy food offerings in posters, leaflets, and media.
3. Staff training regarding healthy eating and allergens
  The chefs, kitchen staff, waiters/waitresses, and all other staff members involved in the restaurant environment received training on nutrition basics, e.g., allergies and intolerance, healthy options and restaurant-recipe ingredients. The training was provided gradually for 2 h/month over 4 sessions.
  The 4 training sessions were basic in nature and had the following themes:
  1. Nutrition and dietetic basics: the food pyramid and nutritional nomenclature.
  2. Types and causes of allergies, including the identification of specific allergens, reactions, differences between allergies and intolerance (such as lactose intolerance) and the associated consequences.
  3. Recipes: the staff were required to know how all dishes were cooked (e.g., ingredients, characteristics, and allergen content).
  4. Communication techniques to inform customers about specific nutrients.

### Outcomes

The principal outcomes included an increased number of healthy dishes on the restaurant menus and an increased offering of allergen-free dishes. The secondary outcome was customer satisfaction with the food.

### Data collection and analysis

The 16 food establishments were assessed as follows:

- The characteristics of the food offered before and at the end of the intervention were recorded in a database according to five groups: gluten-free dishes (all dishes: starters, first courses, second courses and desserts), healthily cooked dishes (starters, first courses and second courses and whether grilled, broiled, or boiled), fried dishes, dishes with vegetables or whole grains (starters, first courses and second courses), and desserts with fruit (desserts only).
- Customer food satisfaction was assessed using a survey on a scale of 0–10 points (0 = worst, and 10 = best).

For the quantitative data, descriptive results were expressed as frequency distributions; continuous data were expressed as the mean ± standard deviation (SD). The differences in dish characteristics before and after the intervention were analysed using McNemar’s test.

To compare the satisfaction of customers, a one-way ANOVA was used. All data were assessed using SPSS software (version 23). The significance level was fixed at a two-sided level of 5%.

## Results

In total, 16 food establishments were assessed (Camping Sangulí: 1 restaurant, 1 takeaway, 1 snack bar and 2 cocktail bars; and the Cambrils Park Resort: 3 restaurants, 1 takeaway, 2 snack bars and 2 cocktail bars). The results were as follows:

1. Analysis of nutritional and allergen contents
  We identified the macronutrient and micronutrient compositions of 360 dishes offered at the restaurants, takeaways and snack bars. The nutritional information for each dish and the additional nutritional labelling are shown in Fig. 1. In addition, the allergen information for each dish was highlighted using stars of different colours (Fig. 1).

**Fig. 1:**
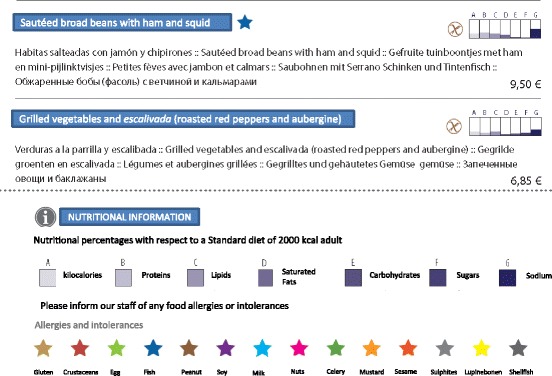
Example of a dish with a description of nutritional values and labelling information
2. Increase in healthy and allergen-free food choices
  2.1) AMED certification was achieved by 3 restaurants across both resorts by implementing changes to avoid contamination with gluten-free food, as shown in Fig. 2. The menus of both resorts met the AMED criteria (see Additional file 1).

**Fig. 2:**
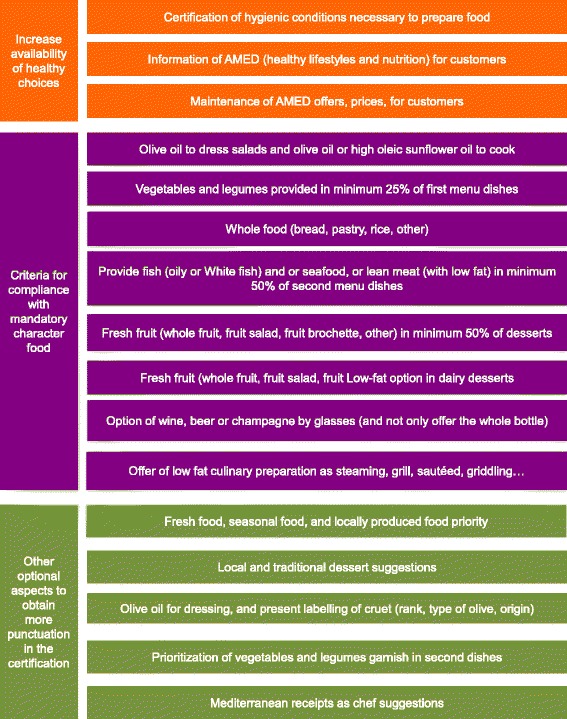
AMED criteria. Translation of AMED criteria [12]
  2.2) In this restaurant-based intervention, specific structural changes were made in the 3 restaurant kitchens at both resorts to facilitate the preparation of gluten-free dishes.
    2.2.1 The chefs from both resorts designed new recipe cards for the gluten-free dishes offered.
    2.2.2 New ovens (1 for each restaurant, takeaway or snack bar) were purchased for exclusive use in preparing gluten-free dishes.
    2.2.3 An external kitchen placed outside of the usual kitchen (1 per restaurant, takeaway or snack bar) was organized for the exclusive preparation of gluten-free and lactose-free dishes.
    2.2.4 Chefs and kitchen staff were newly required to use disposable aprons when preparing gluten-free and/or lactose-free food.
    2.2.5 A specific zone for stocking gluten-free and lactose-free food was established.
    2.2.6 All restaurant, takeaway and snack-bar chefs, waiters/waitresses and other staff received training on the preparation of gluten-free foods and the avoidance of allergen contamination.
  2.3) Many of the dishes offered on the restaurant, takeaway and snack-bar menus could be adapted for lactose-intolerant customers; these dishes were identified by a logo.
  2.4) The 6 restaurant-based strategies were formulated to promote healthy food choices in a family-oriented holiday environment (see Additional file 2).
3. Staff training on healthy eating and allergens
  The chefs, kitchen staff, waiters/waitresses, and all resort staff (355 workers overall) received 4 dietician-led training sessions lasting one hour each in which the specific nutrients and allergens in each dish were identified.

### Dishes offered on the menus of both resorts

From baseline until after the intervention was implemented, there were significant increases in the following types of dishes: gluten-free dishes (2.1% to 50.5%; *P* < 0.001), lactose-free dishes (5.5% to 37.5%; *P* < 0.001), dishes with vegetables (36.2% to 46.9%; *P* = 0.001), healthily cooked dishes (28.6% to 44.7%; *P* = 0.003) and desserts with fruit (20% to 51.3%; *P* = 0.013) (Table 1).

**Table 1:** Changes in the types of dishes offered in restaurants at both resorts before and after the intervention

Dish type	Before intervention (2014)	After intervention (2015)	*P*-value*
	% (n/n total)	% (n/n total)	
Gluten-free dishes^a^	2.1% (7/328)	50.5% (159/315)	**<0.001**
Lactose-free dishes^a^	5.5% (18/328)	37.5% (118/315)	**<0.001**
Dishes with vegetables^b^	36.2% (51/141)	46.9% (61/130)	**0.001**
Fried dishes^b^	24.6 (14/57)	19.6 (10/51)	0.454
Healthily cooked dishes^b^	28.6 (50/175)	44.7 (71/159)	**0.003**
Dishes with whole grains^b^	0% (0/50)	19.2 (10/52)	___
Desserts with fruit^c^	20% (9/45)	51.3 (20/39)	**0.013**

*McNemar’s test

^a^All dishes

^b^Starters, first courses and second courses from the menu

^c^Only desserts

Bold typeface indicates *p*<0.05

Additionally, fresh fruit was introduced as a snack in the 6 bars at both resorts. In the takeaways, fresh fruit was offered in the form of small slices/pieces of various fruit varieties (e.g., watermelon, melon, strawberry, peach, and pineapple) in a lunch box.

The survey results revealed the following regarding the food satisfaction of the resort customers: In 2014, the survey was completed by 20% (*n* = 17,000) of the total customers (*n* = 85,000), with a mean (±SD) of 6.9 ± 1.6 out of 10 points. In 2015, the survey was completed by 23% (*n* = 19,895) of the total customers (*n* = 86,500), with a mean (±SD) of 8.5 ± 1.5 out of 10 points (*P* < 0.001).

In addition, based on the results of our intervention, 4 new strategies emerged, which were added to the 6 strategies proposed by Valdivia Espino et al. [5] to create a 10-strategy restaurant intervention checklist (Table 2).

**Table 2:** Proposal for a checklist of 10 strategies to promote health in restaurant-based interventions

Ten strategies to be considered for promoting health through nutrition in a restaurant-based intervention	Yes	No
Increase the availability of healthy food choices: *increase healthy foods.*		
Enhance the accessibility of healthy food choices: *increase fruit and vegetable options.*		
Reduce prices and improve discount coupons: *provide low-priced, healthy food.*		
Point-of-purchase (POP) information: *provide nutritional labelling on menus.*		
Design restaurant policies: *create policies that incorporate healthy food and healthy preparation techniques.*		
Promote communication: *resort website, television, newspapers, posters, table tents, and other media.*		
Follow healthy preparation practices and healthy menu design based on national recommendations for restaurants (i.e.*, AMED criteria*)*.*		
Follow gluten-free criteria from the celiac association in the country in which the restaurant is located (e.g.*, SMAP criteria*)*.*		
Menu labelling (*allergen and nutritional information*)*.*		
Staff training: *training sessions on nutrition information.*		

## Discussion

The implementation of an intervention in the 16 food-service establishments (restaurants, takeaways and snack bars) of the Cambrils Park Resort and Camping Sangulí significantly increased the offerings of gluten-free dishes (48%), desserts with fruits (31%), dishes including whole grains (19%), healthily cooked dishes (16%) and dishes with vegetables (10%), while the number of fried dishes offered displayed a tendency to decrease. In other words, there was not a significant decrease in the number of fried dishes, which are typical of Spanish gastronomy (e.g., fried fish and certain tapas dishes). However, the quality of the fried dishes increased because the frying oil was changed from vegetable oil to olive oil or high-oleic-acid sunflower oil. This change in oil type represents an important economic challenge because the prices of dishes were maintained.

In this study, increasing the number of dishes that included vegetables and fruits or that were prepared using healthy cooking methods was a way to prevent weight gain [2, 18], and increasing the number and quantity of fruits and vegetables on restaurant menus was a strategy to encourage the consumption of these foods. Thus, greater fruit and vegetable consumption was predicted to have long-term health benefits [19], such as a reduced risk of chronic disease and obesity [20].

To the best of our knowledge, the present study represents the first use of this combination of 6 strategies [5] as a means of promoting health through nutrition implemented in a restaurant-based intervention.

After our intervention, the AMED certificate created by Catalonia’s Public Agency was awarded to the two resorts. The procurement of AMED certification was a challenge because it required increasing the quality and availability of the food. Additionally, the ingredients of the food-service offerings included a high percentage of local, fresh and seasonal food, and all of the changes were adapted without modifying menu prices.

The success of our effort to obtain AMED and SMAP accreditation suggested that the implementation of new policies regarding labelling and other information on menus as well as the regulations that govern the quantity of energy and/or calories and fat provided by dishes aided the design of healthy menus. In addition, in this study, Spain’s new law (*Regulation 1169/2011*) [16] was followed through the provision of attractive visual allergen information for all dishes as an easy means to identify problematic foods for individuals with allergies to and/or intolerance for specific ingredients. Our results were consistent with some of the studies presented in a meta-analysis of 27 restaurant-based nutrition interventions that resulted in the achievement and maintenance of healthy food choices [21].

Labelling nutritional dishes may have a beneficial impact on people’s eating habits outside of the home. However, increasing the number of healthy offerings to promote a beneficial impact on consumer health remains a significant challenge that must be evaluated [22]. Moreover, for individuals who typically eat at restaurants, the influence of the identification of healthy menu strategies as a key factor in increasing their healthy behaviour needs to be corroborated [23].

The health impact results could be the basis for the development of new strategies for the dining environments of food establishments. In our study, food establishments represented a critical means to involve not only individuals but also families in healthy lifestyles at a reasonable price. The cost of dishes is an important factor affecting consumer choice in restaurants [24].

The Centers for Disease Control and Prevention (CDC) proposed additional efforts focused on restaurant recommendations with the aim of addressing the obesity epidemic and obesity-related diseases by encouraging restaurants to offer both reduced- and normal-sized portions [25]. Raising awareness among restaurant owners regarding healthy menus [25], implementing menu labelling, adopting nutrition standards for children’s meals, and promoting healthier meals and food items were the CDC’s future goals. Similarly, in 2006, the U.S. Food and Drug Administration (FDA) recommended the provision of caloric and fat information and an emphasis on low-calorie dishes, among other measures [26]. Thus, the present study contributes to the scarce amount of information on interventions designed to improve healthy food choices in restaurants. In particular, this study helped families manage the diets of family members with an allergy or intolerance by guaranteeing the presence of recipes that were suited to their nutritional needs in a family-oriented holiday environment. Moreover, this restaurant-based intervention represents pioneering work in Spain and provides a checklist of 10 strategies to promote health through nutrition. This checklist of strategies could serve as a step towards a gold standard intervention to support governmental policy tools regarding health and nutrition in food establishments (Table 2).

This study had several limitations, including the small sample of restaurants, snack bars and cocktail bars. One problem faced by restaurant-based interventions is customer demand; thus, the willingness of restaurant owners to increase the number of healthy food choices on their menus was occasionally hindered by customers who preferred fried food.

## Conclusion

In conclusion, this restaurant-based intervention expanded the number of healthy and allergen-free foods offered in a family-oriented holiday resort environment to encourage healthy food choices, resulting in increased customer satisfaction.

## Additional files

Additional file 1: Changes in the kitchen and in the menu of restaurant following AMED criteria. The menus of both resorts were improved following the AMED criteria. Shows the description of these changes in restaurant menus of both resorts and how were achieved. (DOCX 13 kb)

Additional file 2: Six restaurant-based strategies to promote health through nutrition implemented in a restaurant intervention at the Cambrils Park Resort and Camping Sangulí. Shows how were implemented the 6 restaurant-based strategies in restaurants of both resorts. (DOCX 13 kb)

## Abbreviations

AMED: Spanish Mediterranean Diet
FDA: Food and Drug Administration
NEMS-R: Nutrition Environment Measures in Restaurants
SD: Standard deviation
SMAP: Catalan Celiac Association
URV: Universitat Rovira i Virgili
