# Supplementary material for: Restaurant-based intervention to facilitate healthy eating choices and the identification of allergenic foods at a family-oriented resort and a campground
Source: BMC Public Health. 2017 May 5;17:393. doi: 10.1186/s12889-017-4333-5 (PMC5420099; doi:10.1186/s12889-017-4333-5)
Supplement: Supplementary file 1 — Changes in the kitchen and in the menu of restaurant following AMED criteria. The menus of both resorts were improved following the AMED criteria. Shows the description of these changes in restaurant menus of both resorts and how were achieved. (DOCX 13 kb) [file 12889_2017_4333_MOESM1_ESM.docx]

**Additional file 1.** Changes in the kitchen and in the menu of restaurant following AMED criteria.

| AMED Criteria | Before intervention | After intervention. How achieved |
| --- | --- | --- |
| Certification of hygienic conditions necessary to prepare food. | Has already been in place. | Maintained |
| Information of AMED (healthy lifestyles and nutrition) for customers. | X | It has been inserted in each menu. |
| Maintenance of AMED offers, prices, for customers. | X | It has been maintained the prices. |
| Olive oil to dress salads and olive oil or high oleic sunflower oil to cook. | X | For cook, salads and garnish: olive oil.  For fried: high oleic sunflower oil (70% high oleic). |
| Vegetables and legumes provided in minimum 25% of first menu dishes. | X | It has been introduced more than 25% of first menu dishes provided by vegetables and legumes. |
| Whole food (bread, pastry, rice, other) | X | There is the option of whole pasta and rice and whole bread. |
| Provide fish (oliy or White fish) and or seafood, or lean meat (with low fat) in minimum 50% of second menu dishes. | X | Now, we guarantee more than 50% of second menu dishes are based on fish and lean meat. |
| Fresh fruit (whole fruit, fruit salad, fruit brochette, other) in minimum 50% of desserts | X | Now, we guarantee the 50% of desserts are based on fresh fruit. |
| Low-fat option in dairy desserts | X | It has been introduced the low fat option in dairy desserts. |
| Option of wine, beer or champagne by glasses (and not only offer the whole bottle). | X | It can ask for a glass of wine, beer or cava/champagne. |
| Offer of low fat culinary preparation as steaming, grill, sautéed, griddling, etc. | X | We offer low fat culinary preparation. |
| Fresh food, seasonal food, and locally produced food priority. | X | We contact with local producers, change some dishes increasing the fresh food, seasonal food, and locally produced food. |
| Local and traditional dessert suggestions. | Has already been in place. | Maintained and increased. |
| Olive oil for dressing, and present labelling of cruet (rank, type of olive, origin). | Has already been in place. | Maintained |
| Prioritization of vegetables and legumes garnish in second dishes. | X | Now, we offer these criteria. |
| Mediterranean receipts as chef suggestions. | Has already been in place. | Maintained and increased |
| Low fat Mediterranean main dishes in sufficient or half-serving. | X | We offer Mediterranean main dishes and half portion for some dishes. |
| Offer cook dishes without salt and an alternative condiment (pepper or fragrant herbs). | X | We offer the option cook without salt. |
| Free-time active activities dissemination (around the restaurant, routes, etc.). | Has already been in place. | Maintained and increased: participating in Marathon of Priorat, Foodball stay, etc. |
