# Supplementary material for: Restaurant-based intervention to facilitate healthy eating choices and the identification of allergenic foods at a family-oriented resort and a campground
Source: BMC Public Health. 2017 May 5;17:393. doi: 10.1186/s12889-017-4333-5 (PMC5420099; doi:10.1186/s12889-017-4333-5)
Supplement: Supplementary file 2 — Six restaurant-based strategies to promote health through nutrition implemented in a restaurant intervention at the Cambrils Park Resort and Camping Sangulí. Shows how were implemented the 6 restaurant-based strategies in restaurants of both resorts. (DOCX 13 kb) [file 12889_2017_4333_MOESM2_ESM.docx]

**Additional file 2.** Six restaurant-based strategies to promote health through nutrition implemented in a restaurant intervention at the Cambrils Park Resort and Camping Sangulí

| Strategies | How achieved |
| --- | --- |
| Increasing the availability of healthy choices | This strategy increased the availability of number of healthy dishes and healthy preparation techniques that fulfil AMED criteria. The menus now provided more types of fruits and vegetables and healthy preparations, for example, grilled and non-fried options. Dieticians and chefs met to determine menu changes that improved the availability of healthy food (i.e., fruit and vegetable choices), reduced fried options (such as fried potatoes), and increased healthy side-dish options (such as rice, salad, grilled vegetables and mashed potatoes). |
| Raising accessibility of healthy options | This strategy had impulse that restaurant foods used fresh, seasonal, ecological produced food, particularly fruits and vegetables options in main dishes and desserts, provided by local producers while the price of each dish was maintained. |
| Reducing prices to provide healthy and low-priced options | This strategy increased the variety and quality of menu choices while maintaining adequate menu prices. |
| Informing consumers regarding the nutritional value of the dishes offered on restaurant menus (POP information) | This strategy was oriented to identify allergens for each dish on the menu used specific labelling, created by us. In addition, we calculated nutritional value percentages and introduced nutritional labelling for each dish on the menu in the restaurants and snack bars. Specifically, the lowest-calorie menu dish was labelled with a heart image. Furthermore, the waiters/waitresses received training regarding nutritional (i.e., allergic and nutrition) issues to be able to provide suitable information to customers, if it was required. |
| Designing restaurant policies | This strategy was focused, for example, regarding healthy cooking techniques.  AMED and SMAP certificates were obtained. |
| Promoting and communicating healthy food offerings | This strategy was a key point to inform healthy dishes to costumers by posters, leaflets, and media. Healthy lifestyle recommendations were placed on the resorts website.  For example, Mediterranean Diet food-group pyramid was displayed in the restaurants and the chef’s menu suggestions were based on Mediterranean recipes. Various television channels and newspapers informed the novelties offered in food establishments of the resorts. |

POP = point-of-purchase; AMED = Mediterranean Diet certification (Spanish government); SMAP = Catalan Celiac Association.
